# Short-Chain Fatty Acid and FFAR2 Activation – A New Option for Treating Infections?

**DOI:** 10.3389/fcimb.2021.785833

**Published:** 2021-12-02

**Authors:** Katja Schlatterer, Andreas Peschel, Dorothee Kretschmer

**Affiliations:** ^1^ Infection Biology, Interfaculty Institute for Microbiology and Infection Medicine Tübingen (IMIT), University of Tübingen, Tübingen, Germany; ^2^ German Center for Infection Research, Partner Site Tübingen, Tübingen, Germany; ^3^ Cluster of Excellence Cluster of Excellence (EXC) 2124 Controlling Microbes to Fight Infections, University of Tuebingen, Tübingen, Germany

**Keywords:** short-chain fatty acids, GPR43/FFAR2, infectious diseases, antimicrobial resistance, therapeutic application, multidrug resistant infections

## Abstract

The human innate immune system is equipped with multiple mechanisms to detect microbe-associated molecular patterns (MAMPs) to fight bacterial infections. The metabolite short-chain fatty acids (SCFAs) acetate, propionate and butyrate are released by multiple bacteria or are food ingredients. SCFA production, especially acetate production, is usually essential for bacteria, and knockout of pathways involved in acetate production strongly impairs bacterial fitness. Because host organisms use SCFAs as MAMPs and alter immune reactions in response to SCFAs, interventions that modulate SCFA levels can be a new strategy for infection control. The interaction between SCFAs and host cells has been primarily investigated in the intestinal lumen because of the high local levels of SCFAs released by bacterial microbiome members. However, members of not only the intestinal microbiome but also the skin microbiome produce SCFAs, which are known ligands of the seven-transmembrane G-protein-coupled receptor FFAR2. In addition to enterocytes, FFAR2 is expressed on other human cell types, including leukocytes, especially neutrophils. This finding is in line with other research that determined that targeted activation of FFAR2 diminishes susceptibility toward various types of infection by bacteria such as *Klebsiella pneumonia, Citrobacter rodentium*, and *Staphylococcus aureus* but also by viruses such as respiratory syncytial and influenza viruses. Thus, our immune system appears to be able to use FFAR2-dependent detection of SCFAs for perceiving and even averting severe infections. We summarize recent advances in understanding the role of SCFAs and FFAR2 in various infection types and propose the manipulation of this receptor as an additional therapeutic strategy to fight infections.

## Introduction

The World Health Organization (WHO) proclaimed the emergence of multiresistant pathogens to be a major threat to human health, urging the development of alternative approaches to the use of antibiotics for the prevention and treatment of infectious diseases (https://www.who.int/news-room/fact-sheets/detail/antimicrobial-resistance). One such approach could be enhancing the antimicrobial capacity of leukocytes, leading to enhanced elimination of pathogens. As a hallmark of the presence of invading pathogens, the innate immune system uses a variety of pattern recognition receptors (PRRs) to detect conserved microbe-associated molecular pattern molecules (MAMPs). PPRs that belong to the group of Toll-like (TLRs), nucleotide-binding oligomerization domain-like (NLRs) and formyl-peptide (FPR) receptors are necessary for the recognition of various bacterial MAMPs, such as lipopeptides, lipopolysaccharides, cell wall fragments and formylated peptides (Coll and O'Neill, 2010; Bloes et al., 2015). However, bacteria-derived metabolites such as short-chain fatty acids (SCFAs) can also be regarded as MAMPs. The SCFAs acetate, propionate and butyrate have been identified as ligands of the seven-transmembrane G-protein-coupled receptors (GPCRs) FFAR2 (former GPR43) and FFAR3 (former GPR41) (Le Poul et al., 2003; Nohr et al., 2013). The interaction between SCFAs and host cells has been mainly analyzed in the intestinal lumen (Goncalves et al., 2018). However, we are now beginning to understand how SCFAs also modulate the function of innate immune cells such as neutrophils, monocytes or macrophages in other tissues and in the blood (Ang and Ding, 2016). Intriguingly, activation of FFAR2 by SCFA administration, especially acetate, diminishes the susceptibility toward various types of infections caused by bacteria and viruses (Galvao et al., 2018; Antunes et al., 2019; Sencio et al., 2020). A common feature of such SCFA-mediated processes appears to be the direct or indirect activation of leukocytes through FFAR2 stimulation. To highlight these new discoveries, this review will provide an outline of the current knowledge about FFAR2 activation and a perspective on how this knowledge could be used for the treatment of infectious diseases.

### Production of SCFAs Under Aerobic and Anaerobic Conditions by Members of the Gut and Skin Microbiota

**Figure 1:**
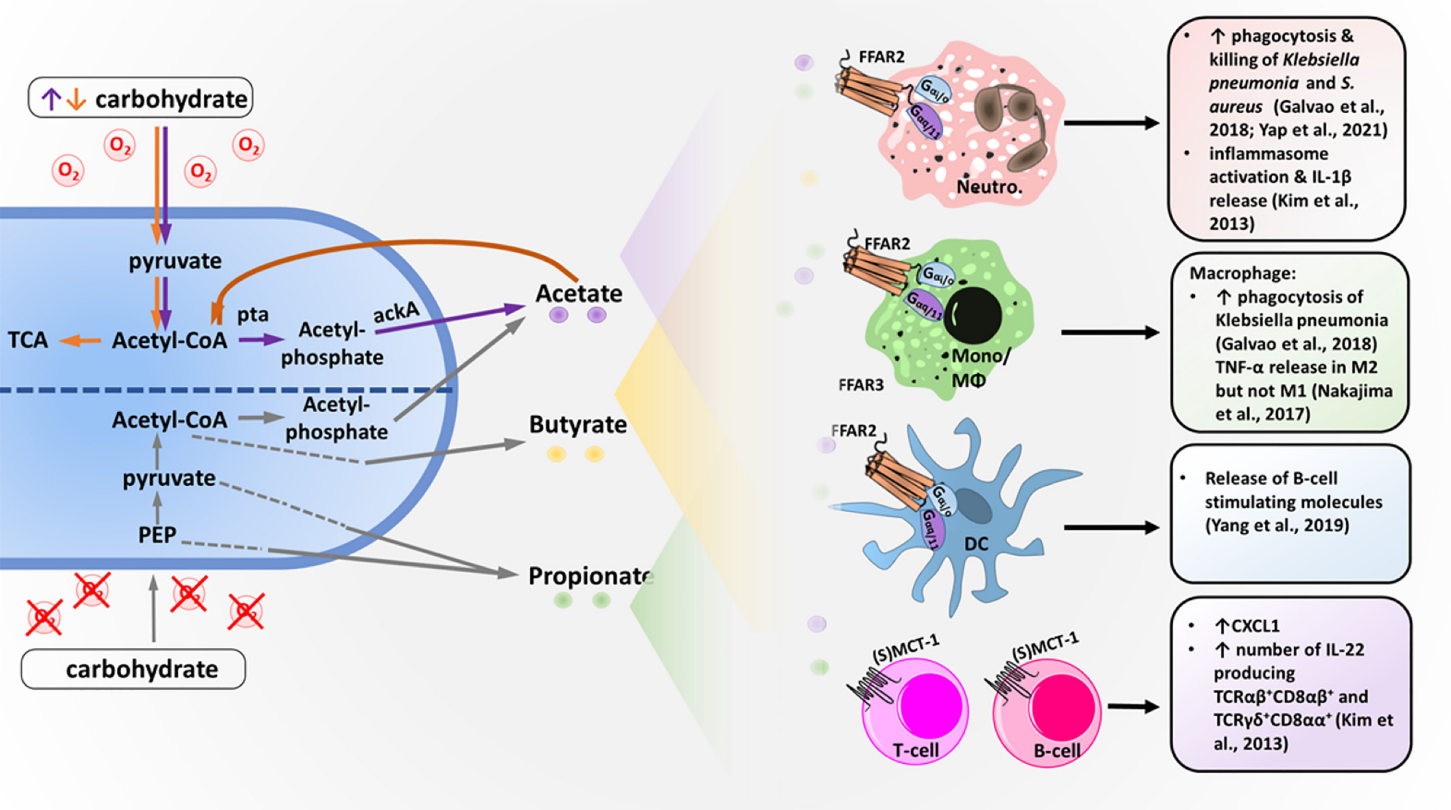
Bacterial short-chain fatty acid (SCFA) production and SCFA effects on different immune cells. Under anaerobic conditions, bacteria metabolize carbohydrates into SCFA acetate, propionate and butyrate using different pathways. Acetate and butyrate are primarily produced *via* acetyl-CoA, whereas propionate is made from pyruvate or phosphoenolpyruvat (PEP) *via* multiple different pathways. Under aerobic conditions and excess carbohydrates (orange arrows), carbohydrates are digested into acetate *via* acetyl-CoA using the phosphatase/acetyl-kinase A (Pta/AckA) pathway. Acetate, butyrate and propionate are secreted into the bacterial environment and can then be detected by different immune cells. Neutrophils (Neutro.), monocytes (Mono.), macrophages (MΦs) and dendritic cells (DCs) express the SCFA receptor FFAR2, which is coupled with the G-protein G-alpha i/o and G-alpha q/11. T and B cells lack FFAR2 but express the acetate transporter (sodium-) monocarboxylate transporter 1 ((S)MCT-1), which allows acetate to modulate transcription *via* histon deacetylase stimulation. Activation of the different immune cells by SCFAs results in the effects described on the right.

It has been known for a long time that bacterial metabolites from the intestinal microbiota can influence the human host locally as well as systemically, resulting in the modulation of inflammatory reactions (Goncalves et al., 2018). The best-studied intestinal bacterial metabolites that influence inflammation are SCFAs, which are organic carboxylic acids that contain aliphatic backbones with one to six carbon atoms (Tan et al., 2014). High concentrations of SCFAs can be detected in the human intestine, where they are the primary end-products of anaerobic fermentation by gut bacteria. The most frequent SCFAs found in the intestine are acetate (C2), followed by propionate (C3) and butyrate (C4), with an average ratio of approximately 60-20-20 (Cummings et al., 1987; Boets et al., 2017). Bacterial SCFA production can be the result of different fermentation pathways (Alexander et al., 2019). Under the anaerobic conditions of the gut, the glycolysis product pyruvate can be converted to acetyl-CoA and further hydrolyzed to acetate or (via butyric fermentation) hydrolyzed to butyryl-CoA followed by butyrate production. For pyruvate production, various other pathways in addition to glycolysis are used (Louis et al., 2014; Morrison and Preston, 2016). Additionally, under aerobic conditions, some bacteria, including *Staphylococcus aureus*, an important pathogen frequently involved in skin and wound infections, produce high concentrations of acetate *via* the Pta-AckA pathway (Sadykov et al., 2013; Marshall et al., 2016; Won et al., 2021). This pathway is utilized for energy production under carbon overflow and subsequent citric acid cycle blockage. The produced SCFAs can be taken up by epithelial cells either passively or, more often, actively *via* monocarboxylate transporter 1 (MCT1) or sodium-coupled monocarboxylate transporter 1 (SMCT-1) (**Figure 1**) (Halestrap and Meredith, 2004). Transport of nonused SCFAs out of the human cell most likely occurs *via* further unknown pathways.

For nonintestinal microbiota, the abundance of major bacterial metabolites and their immunomodulatory functions are still largely unknown (Byrd et al., 2018). For the skin and nasal commensal *Cutibacterium acnes* (previously known as *Propionibacterium acnes*), the production of butyrate, valerate and propionate has been observed under hypoxic conditions. This process amplifies TLR responsiveness and enhances proinflammatory cytokine expression (Sanford et al., 2016; Sanford et al., 2019), but it has remained unclear whether it depends on FFAR2 or FFAR3. *S. aureus* and the skin and nasal commensals *Staphylococcus epidermidis* and *Staphylococcus hominis* produce high amounts of SCFA acetate (**Figure 1**) (Lam et al., 2018). However, whether these SCFAs exacerbate or mitigate inflammation on skin or during invasive infection is still controversial in the field (Ang and Ding, 2016; Parada Venegas et al., 2019). In addition to microbiota metabolism, food intake and the liver represent potential sources of acetate. The liver produces acetate under certain conditions, such as starvation or diabetes (Tang and Offermanns, 2017).

### Increase in SCFA Concentrations During Various Types of Infectious Diseases

Metabolic end-products that are produced from not only commensal but also invading bacteria can influence host cells, thereby potentially affecting the course of an infection. To grow and produce virulence factors, invading pathogens depend on host molecules for energy generation. Due to the consumption of O_2_ and nutrients by immune cells during immune reactions, infection sites such as abscesses are often hypoxic, and only limited amounts of the primary carbon source glucose are available (Eltzschig and Carmeliet, 2011; Kedia-Mehta and Finlay, 2019). This circumstance limits bacterial energy generation through glycolysis and the citric acid cycle (TCA), resulting in the utilization of secondary carbon sources such as amino acids (Morrison and Preston, 2016) and leading to SCFA production. Analysis of infection sites and abscesses revealed that they harbor considerably high concentrations of SCFAs, especially acetate (Ladas et al., 1979; Fanos et al., 2014; Brook, 2016; Lussu et al., 2017). This situation could be shown for abscesses, periodontitis, urinary tract infections or vaginosis caused by various different bacteria (Ladas et al., 1979; Tonetti et al., 1987; Chaudry et al., 2004; Lu et al., 2014; Aldunate et al., 2015; Lussu et al., 2017).

Additionally, generalized infections such as bacteremia often co-occur with increased serum acetate concentrations, which has been found in different septic animal models (Fanos et al., 2014; Balmer et al., 2016). Interestingly, treatment of some of these infections was associated with a drastic reduction in serum SCFA levels, which points to bacteria as the main source of SCFAs (Chaudry et al., 2004; Qiqiang et al., 2012; Lu et al., 2014; Jorth et al., 2014). This finding is supported by a recent study, which showed that the oral commensal Fusobacterium nucleatum, implicated in periodontal diseases, releases high amounts of SCFAs (Dahlstrand Rudin et al., 2021). However, as a consequence of catabolic and metabolic stress, increased SCFA release from host tissues has also been suspected (Balmer et al., 2016). Host cells such as hepatocytes are equipped with an acetyl-CoA hydrolase, converting acetyl-CoA into free acetate, which represents a rapid energy source for energy-starved tissues (Knowles et al., 1974; Buckley and Williamson, 1977). Independent of their origin, SCFAs appear to stimulate leukocytes, which could subsequently influence the course of an infection.

### Interaction of SCFAs With Host Innate Immune Cells *via* FFAR2

SCFAs, released either by commensal or pathogenic bacteria, can contribute to the modulation of inflammatory responses (Ratajczak et al., 2019). Direct modulation can be mediated by SCFA-specific surface receptors, the so-called free fatty acid receptors (FFARs), which belong to FFAR3 and FFAR2 (Milligan et al., 2017). In 2003, two independent groups discovered SCFAs to be ligands for the seven-transmembrane GPCRs FFAR2 and FFAR3 (Brown et al., 2003; Le Poul et al., 2003). Their SCFA-binding affinity ranges from high micromolar to low millimolar concentrations, which makes them only moderately sensitive to SCFAs compared to other GPCR ligands. Low sensitivity might prevent hyperactivation of these receptors. Among the different SCFAs, acetate and propionate are the preferred ligands for FFAR2 (EC_50_ of 250-500 μM) (40), whereas FFAR3 preferably binds butyrate (Bolognini et al., 2016; Milligan et al., 2017). Under healthy conditions, acetate reaches a mean concentration of 25-100 μM in venous blood (Cummings et al., 1987; Hoving et al., 2018). Outside of the gut, only acetate can reach the high concentrations needed to activate FFAR2 under physiological or pathological conditions.

In humans, FFAR2 is highly expressed on the surface of leukocytes, especially neutrophils (Brown et al., 2003; Le Poul et al., 2003), but it is also found on dendritic cells, monocytes, enterocytes, pancreatic β-cells and adipocytes (Hong et al., 2005; Nohr et al., 2013; McNelis et al., 2015; Yang et al., 2019). In monocytes, FFAR2 is expressed at low levels, whereas in lymphocytes, no expression of SCFA-recognizing receptors was observed (Le Poul et al., 2003; Ang and Ding, 2016; Milligan et al., 2017; Parada Venegas et al., 2019). In addition, mucosal mast cells in the rat intestine express FFAR2 (Karaki et al., 2006). Moreover, various research groups found FFAR2 to be upregulated by different MAMPs, such as LPS, and by SCFAs themselves (Ang et al., 2015; Nakajima et al., 2017). Among the SCFAs, acetate and propionate are the preferred ligands for FFAR2, whereas FFAR3 preferably binds butyrate and propionate but is 10-fold less sensitive to acetate. In the intestinal lumen, the most frequently found SCFA is acetate, followed by propionate and butyrate, with a ratio of 60-20-20 (Cummings et al., 1987; Boets et al., 2017). The SCFA ratio shifts in healthy venous blood to 90-5-5 for acetate, propionate and butyrate (Cummings et al., 1987; Hoving et al., 2018). Therefore, activation of FFARs outside of the human gut is most likely largely driven by acetate. Which human cell type expresses FFAR3 is, however, less clear and sometimes controversial. FFAR3 is thought to be mainly expressed on enteroendocrine cells as well as on neurons (Nohr et al., 2013), but some groups also proposed low-level FFAR3 expression by peripheral blood mononuclear cells (Brown et al., 2003; Maslowski et al., 2009).

Combined with high expression in neutrophils, this upregulation strongly implies an involvement of FFAR2 and SCFAs in infection control. This assumption is further supported by the finding that FFAR2 was associated with inflammatory gene expression networks. Nearest-neighbor correlation analysis of transcriptional profiles revealed that the expression of FFAR2 is coregulated with that of PRRs such as TLR2, FPR1 and FPR2 and can thus be considered to be part of an inflammatory network cluster (Maslowski et al., 2009). Additionally, transcriptomic analysis of neutrophils from septic patients showed that the expression of the *ffar2* gene follows that of other inflammatory genes and is dysregulated during sepsis (Godini et al., 2018). FFAR2 appears to be able to initiate two different intracellular pathways by binding two different small G-proteins, at least in neutrophils. The G-proteins Gα_q_ and Gα_i/o_ show affinity to the N-terminal G-protein binding site of FFAR2 (Brown et al., 2003), which is known to initiate different signaling cascades. Gα_i/o_-dependent pathway activation results in intracellular accumulation of cAMP as well as activation of the phospholipase C (PLC) pathway and an increase in intracellular calcium levels (Kamato et al., 2015). Additionally, signaling cascades result in phosphorylation of the MAP kinases (MAPK), ERK1/2 and p38, which then modulate diverse effector functions in cells (Vinolo et al., 2011; Kamato et al., 2015).

The best described and established effector function of FFAR2 activation in neutrophils is the induction of chemotaxis toward local sites of infection or inflammation (Le Poul et al., 2003; Maslowski et al., 2009), whereas the effect on other neutrophil functions, such as the release of cytokines, is less clear. Additionally, controversial data were reported for FFAR2-dependent cytokine and antimicrobial peptide release, with either stimulatory or inhibitory effects (Ang and Ding, 2016). Therefore, it remains unclear whether FFAR2 activation has pro- or anti-inflammatory consequences.

### SCFAs as Immune Modulators During Infection

Histone deacetylases (HDACs) influence the degree of acetylation of histones and nonhistone proteins and thereby influence overall transcription. Most receptor-independent effects of SCFAs, especially butyrate and propionate, were found to be anti-inflammatory and mediated by inhibition of HDACs. Since histone deacetylases are expressed in endothelial cells, butyrate and propionate reduce the expression of proinflammatory genes and prevent endothelial leakage (Paulus et al., 2011). Furthermore, SCFCs enhance the expression of epithelial barrier forming molecules and mucin production (Burger-van Paassen et al., 2009). This effect can positively influence inflammatory disorders, e.g., sepsis. It was assumed that the HDAC inhibitory activity of butyrate increases cellular infection with viruses, including influenza virus, reovirus, and HIV-1, because of the suppression of specific antiviral IFN-stimulated gene (ISG) products (Chemudupati et al., 2020). However, acetate has no inhibitory effect on HDACs but induces strong FFAR2-dependent signaling (Kendrick et al., 2010). Although increased concentrations of SCFAs, especially acetate, are observed in various types of infections, little is known about the role of SCFAs and FFAR2 during infectious diseases. In 2003, Le Poul proposed a role of FFAR2 in infection control (Le Poul et al., 2003). Chronic and acute alcohol abuse is characterized by an increased susceptibility to infections (Corberand et al., 1989; Shellito et al., 2001) and an impaired inflammatory response (Todorovic et al., 1994; Todorovic et al., 1999) as well as decreased neutrophilic bactericidal and chemotactic capacity (Laharrague et al., 1985; Bautista, 2002). Serum acetate levels can increase up to 1 to 2 mM after alcohol consumption due to conversion of ethanol to acetate by liver enzymes (Nuutinen et al., 1985; Peng et al., 2007; Jiang et al., 2013). Le Poul hypothesized that some of these effects could be explained by FFAR2 desensitization as a consequence of high concentrations of acetate in the serum, which would subsequently impair the migration of neutrophils to the site of a bacterial infection (Le Poul et al., 2003).

More recently, some research groups have investigated the role of FFAR2 in infections in more detail using FFAR2 knockout mice or FFAR2 inhibitors. They showed that FFAR2-expressing dendritic cells (DCs), as well as epithelial cells, are involved in the antibody response against cholera toxin and *Citrobacter rodentium* (**Table 1**) (Kim et al., 2013; Yang et al., 2019; Yap et al., 2021). Furthermore, the combination of acetate and butyrate facilitated the induction of antigen-specific IgA and IgG responses after oral cholera toxin immunization. Thus, SCFAs were proposed to be useful as adjuvants (Yang et al., 2019). Consistently, due to a decrease in the antibody response, FFAR2 knockout mice showed a higher susceptibility to *C. rodentium* infection than wild-type mice (Yang et al., 2019). One year earlier, FFAR2 knockout mice were found to be more susceptible to lung infection by *Klebsiella pneumonia* (Galvao et al., 2018). The expression of FFAR2 on neutrophils and alveolar macrophages proved to be important for the clearance of *K. pneumonia* from infected lungs. Increased acetate concentrations in drinking water ameliorated infection outcomes by increasing the phagocytic capacity of neutrophils and macrophages (**Table 1**) (Galvao et al., 2018). During *Clostridium difficile* infection, FFAR2 signaling was shown to accelerate neutrophil recruitment *via* enhanced production of CXCL1, to activate the inflammasome and to augment IL-1 receptor expression as well as IL-22 secretion by innate lymphocytes (Fachi et al., 2020).

**Table 1:** Consequences of the treatment of infectious diseases with the FFAR2 ligand acetate.

	Infection model	Targeted cells/organs/effect	Treatment	Outcome
(Antunes et al., 2019)	**Pulmonary infection.** WT and FFAR2^−/−^ mice infected with **respiratory syncytial virus (RSV)**	Activation of **murine pulmonary epithelial cells *via*** FFAR2 promoted antiviral effects through an IFN-β response.	**Four-week high fiber diet** prior and during RSV infection. Or **SCFA- drinking water (200 mM**) for 3 weeks prior RSV infection	Acetate treatment protects against RSV infection.
(Bautista, 2002)	**Gut infection** of mice with ***Clostridium difficile***	FFAR2 signaling in **neutrophils** and in **ILC3s**	Acetate **(150 mM) administered in drinking water** before infection	Microbiota-derived acetate coordinates action on neutrophils and ILC3s in response to *C. difficile*
(Galvao et al., 2018)	**Pulmonary infection.** WT and FFAR2^-/-^ mice infected **with *Klebsiella pneumoniae***	FFAR2 expression, especially in **neutrophils and alveolar macrophages,** is important for bacterial phagocytosis and killing.	Acetate **(150 mM)** added to the **drinking water of mice**	Acetate treatment leads to reduced bacterial numbers in the airways
(Sencio et al., 2020)	**Pulmonary infection.** Infection with influenza A virus and *S. pneumoniae* superinfection.	Reduced production of acetate affects the bactericidal activity of **alveolar macrophages.**	Acetate (**200 mM) added to the drinking water five days before the** *S. pneumoniae* challenge.	FFAR2 activation during influenza reduces bacterial superinfection
(Todorovic et al., 1994)	**Gut infection.** WT, FFAR3^-/-^ and FFAR2^-/-^ mice infected with ***Citrobacter rodentium***	Acetate administration accelerated IL6, CXCL1/2 expression in **epithelia cells** and **neutrophil/Th17** recruitment in the large cecum	Acetate (200 mM) **added to** the drinking water for 4 weeks	Acetate-fed WT mice suffered less than untreated mice from infection
(Todorovic et al., 1999)	**Gut infection.** WT and FFAR2^-/-^ mice infected with ***Citrobacter rodentium***	Upon acetate treatment, numbers of colonic IL-22 producing **intraepithelial lymphocytes** are increased.	Fed with high acetate diet *ad libitum* for 3 weeks prior and during infection	High SCFA‐producing diets affected infection in mice: less pathogens and altered gut microbiota **composition**
(Corberand et al., 1989)	**Gut infection *Citrobacter rodentium*** infection of WT and FFAR2^−/−^ mice	Acetate and butyrate promote B-cell IgG production and plasma cell differentiation-related genes through interaction with **FFAR2 on dendritic cells.**	Oral immunization with Ovalbumin and cholera toxin. A mixture of acetate/butyrate (300 mM) was added to drinking water containing antibiotics for 28 d.	SCFA administration promoted intestinal antibody responses in WT mice
(Laharrague et al., 1985)	**Bacteremia, peritonitis *Staphylococcus aureus*** infection of WT and FFAR2^−/−^ mice	Acetate primed neutrophils in a FFAR2-dependent fashion, leading to enhanced neutrophil oxidative burst and bacterial killing.	i.p. injection of 500 mg/kg acetate prior (30 min) or post (6 h) sepsis induction or addition of (150 mM) acetate to drinking water for 5 days.	In WT mice, acetate administration reduced bacterial numbers in peripheral organs by several magnitudes

SCFAs, Short-chain fatty acids; RSV, respiratory syncytial virus; WT, wild-type; OVA, ovalbumin; ILC3s, type 3 innate lymphoid cells; i.p., intraperitoneal.

In 2019, Antunes et al. found that acetate treatment of mice improves not only the outcome of bacterial infections but also the outcome of certain viral infections (Antunes et al., 2019). Nasal application of acetate reduced the viral load and pulmonary inflammation after respiratory syncytial viral infection in an FFAR2-dependent manner. A high-fiber diet could mimic this effect by enhancing SCFA production by gut microbes, an effect that was abolished by antibiotic treatment (Antunes et al., 2019). Treatment with acetate during infection by influenza A virus and by *Streptococcus pneumoniae* superinfection improved the survival rates of double-infected mice (Sencio et al., 2020). In addition, we recently showed that elevated serum acetate concentrations prime and alert neutrophils in an FFAR2-dependent fashion. This priming improved the capacity of human neutrophils to eliminate methicillin-resistant *S. aureus* (MRSA) and rescued wild-type but not FFAR2-/- mice from severe *S. aureus* sepsis (Schlatterer et al., 2021).

In addition to these results, which are based on mouse studies, the first clinical observations propose an involvement of FFAR2 in human infectious diseases, as outlined in the next section (Carr et al., 2018). In summary, all of these different findings (**Table 1**) suggest a relevant role of changing SCFA concentrations and FFAR2 during human infectious diseases.

### FFAR2 as a Possible Pharmaceutical Target for Modulating Infectious Diseases

A recent clinical study revealed that septic patients with an elevation in whole-blood FFAR2 receptor expression had a significantly increased 30-day survival (Carr et al., 2018). Therefore, FFAR2 modulation by synthetic or even natural ligands could be a beneficial therapeutic option for improved infection control. A change in dietary habits could increase SCFA levels in the human circulation and could positively influence an infection outcome. The microbiota composition also influences SCFA levels in the circulation, and different bacterial species are known to produce more SCFAs than others (Agus et al., 2016). Bacteria of the phylum Bacteroides are strong SCFA producers and could be beneficial during viral infections, since Bacteroidetes produce high levels of acetate and propionate, whereas Firmicutes produce more butyrate (Macfarlane and Macfarlane, 2003). However, in contrast to the rare propionate and butyrate production pathways (Louis et al., 2010), pathways for acetate production are widely distributed among bacterial groups and in many Firmicutes. Thus, probiotic alteration of the composition of the microbiome could influence infection susceptibility.

The idea of manipulating FFAR2 during an infectious disease is quite intriguing. However, we need to elucidate further the exact immunomodulatory effect of acetate; the reason is that while favorable during an infection, an increased immune reaction could worsen chronic inflammatory diseases such as inflammatory bowel diseases (IBDs). For the treatment of IBD ulcerative colitis, Galapagos tested a human FFAR2-specific antagonist called GLPG0974 in phase I and II clinical trials. However, the development of GLPG0974 was stopped since the expected clinical endpoints were not achieved. Nonetheless, GLPG0974 was able to reduce neutrophil activation and infiltration into inflamed tissue (Suckow and Briscoe, 2017), showing that FFAR2 manipulation of human leukocytes might have effects in human patients. Although a general activation of FFAR2 might be useful to fight infections, it could have multiple side effects due to the implication of FFAR2 in other physiological processes, including metabolic and brain functions (Milligan et al., 2017; Chambers et al., 2018; Dalile et al., 2019). Nevertheless, for life-threatening infections such as severe sepsis, transient FFAR2 manipulation could represent a novel therapeutic strategy for boosting immune reactions and improving the outcome of infectious diseases.

## Conclusions

A common feature of many infectious diseases is a local or systemic increase in the amounts of SCFAs, especially acetate (Gorbach et al., 1976; Aldunate et al., 2015). In recent years, SCFAs were reported to interact with leukocytes *via* receptor-dependent and receptor-independent immune modulation. Especially for butyrate, FFAR2-independent inhibition of histone deacetylases (HDACs) has been described, which downregulates transcription and thereby also influences inflammation. In contrast to butyrate, no HDAC inhibition has been described for acetate (Waldecker et al., 2008; Sanford et al., 2016), which is the preferred ligand of FFAR2 (Milligan et al., 2017). Therefore, the use of different members of the SCFA family as well as different models might be the reason for the controversial findings that concern the effect of SCFAs on local inflammation (Nadeem et al., 2017; Krejner et al., 2018). Additionally, in contrast to classical PRRs such as FPRs, the consequences of downstream FFAR2 signaling are rather ambiguous. FFAR2 is coupled to two different Gα subunits (Brown et al., 2003). The sole activation of Gαi-coupled receptors such as FPRs leads to directed migration of neutrophils. However, it is unclear whether activation of Gαi/Gαq-coupled FFAR2 leads directly or only indirectly, e.g., *via* chemokine induction or amplification of other signals, to neutrophil migration. We suspect that differential activation of the G-protein alpha-subunits could be a reason for the varying findings regarding inflammation and migration. In addition, the correlation of FFAR2 expression with the expression of PRRs, such as TLR2 or FPR1, indicates that FFAR2 expression could influence the activation of these receptors (Godini et al., 2018). All of these concerns might contribute to the fact that the exact mechanism as well as the outcome of SCFA-FFAR2 modulation is currently controversial.

Over the past few years, an increasing number of publications have shown a positive effect of direct or indirect acetate administration on infections. For example, a high-fiber diet leading to SCFA generation in the gut or oral administration of acetate influences the outcome of viral and bacterial infections (**Table 1**). Even manipulation of the gut microbiota has been shown to influence the amount of SCFAs released in the circulation. Therefore, the idea of targeted SCFA administration during infectious disease or the intake of a high-fiber diet to enhance microbiome SCFA production could be a strategy to treat and prevent such infections.

More studies with human leukocytes, human tissue and especially clinical studies are needed. An improved immune reaction for amended infection control could be a new antimicrobial and antiviral approach in times of emerging antibiotic resistance.

## Author Contributions

KS, AP and DK wrote and reviewed the manuscript. KS prepared the figure. DK and KS summarized the tables. All authors contributed to the article and approved the submitted version.

## Funding

This study was funded by grants from the German Research Foundation (SFB685 to AP and TRR34 and TR156, project ID 246807620, to DK and AP) and the German Center for Infection Research (DZIF) to AP and DK. The authors acknowledge infrastructural support by the Cluster of Excellence EXC2124 Controlling Microbes to Fight Infections, project ID 390838134.

## Conflict of Interest

The authors declare that the research was conducted in the absence of any commercial or financial relationships that could be construed as a potential conflict of interest.

## Publisher’s Note

All claims expressed in this article are solely those of the authors and do not necessarily represent those of their affiliated organizations, or those of the publisher, the editors and the reviewers. Any product that may be evaluated in this article, or claim that may be made by its manufacturer, is not guaranteed or endorsed by the publisher.

## References

[B1] Agus A. Denizot J. Thevenot J. Martinez-Medina M. Massier S. Sauvanet P.. (2016). Western Diet Induces a Shift in Microbiota Composition Enhancing Susceptibility to Adherent-Invasive E. Coli Infection and Intestinal Inflammation. Sci. Rep. 6, 19032. doi: 10.1038/srep19032 26742586 PMC4705701

[B2] Aldunate M. Srbinovski D. Hearps A. C. Latham C. F. Ramsland P. A. Gugasyan R.. (2015). Antimicrobial and Immune Modulatory Effects of Lactic Acid and Short Chain Fatty Acids Produced by Vaginal Microbiota Associated With Eubiosis and Bacterial Vaginosis. Front. Physiol. 6, 164. doi: 10.3389/fphys.2015.00164 26082720 PMC4451362

[B3] Alexander C. Swanson K. S. Fahey G. C. Garleb K. A. (2019). Perspective: Physiologic Importance of Short-Chain Fatty Acids From Nondigestible Carbohydrate Fermentation. Adv. Nutr. 10, 576–589. doi: 10.1093/advances/nmz004 31305907 PMC6628845

[B4] Ang Z. Ding J. L. (2016). GPR41 and GPR43 in Obesity and Inflammation - Protective or Causative? Front. Immunol. 7, 28. doi: 10.3389/fimmu.2016.00028 26870043 PMC4734206

[B5] Ang Z. Er J. Z. Ding J. L. (2015). The Short-Chain Fatty Acid Receptor GPR43 Is Transcriptionally Regulated by XBP1 in Human Monocytes. Sci. Rep. 5, 8134. doi: 10.1038/srep08134 25633224 PMC4311239

[B6] Antunes K. H. Fachi J. L. de Paula R. da Silva E. F. Pral L. P. Dos Santos A. A.. (2019). Microbiota-Derived Acetate Protects Against Respiratory Syncytial Virus Infection Through a GPR43-Type 1 Interferon Response. Nat. Commun. 10, 3273. doi: 10.1038/s41467-019-11152-6 31332169 PMC6646332

[B7] Balmer M. L. Ma E. H. Bantug G. R. Grahlert J. Pfister S. Glatter T.. (2016). Memory CD8(+) T Cells Require Increased Concentrations of Acetate Induced by Stress for Optimal Function. Immunity 44, 1312–1324. doi: 10.1016/j.immuni.2016.03.016 27212436

[B8] Bautista A. P. (2002). Acute Ethanol Binge Followed by Withdrawal Regulates Production of Reactive Oxygen Species and Cytokine-Induced Neutrophil Chemoattractant and Liver Injury During Reperfusion After Hepatic Ischemia. Antioxid. Redox Signal 4, 721–731. doi: 10.1089/152308602760598864 12470499

[B9] Bloes D. A. Kretschmer D. Peschel A. (2015). Enemy Attraction: Bacterial Agonists for Leukocyte Chemotaxis Receptors. Nat. Rev. Microbiol. 13, 95–104. doi: 10.1038/nrmicro3390 25534805

[B10] Boets E. Gomand S. V. Deroover L. Preston T. Vermeulen K. De Preter V.. (2017). Systemic Availability and Metabolism of Colonic-Derived Short-Chain Fatty Acids in Healthy Subjects: A Stable Isotope Study. J. Physiol. 595, 541–555. doi: 10.1113/JP272613 27510655 PMC5233652

[B11] Bolognini D. Tobin A. B. Milligan G. Moss C. E. (2016). The Pharmacology and Function of Receptors for Short-Chain Fatty Acids. Mol. Pharmacol. 89, 388–398. doi: 10.1124/mol.115.102301 26719580

[B12] Brook I. (2016). Spectrum and Treatment of Anaerobic Infections. J. Infect. Chemother. 22, 1–13. doi: 10.1016/j.jiac.2015.10.010 26620376

[B13] Brown A. J. Goldsworthy S. M. Barnes A. A. Eilert M. M. Tcheang L. Daniels D.. (2003). The Orphan G Protein-Coupled Receptors GPR41 and GPR43 Are Activated by Propionate and Other Short Chain Carboxylic Acids. J. Biol. Chem. 278, 11312–11319. doi: 10.1074/jbc.M211609200 12496283

[B14] Buckley B. M. Williamson D. H. (1977). Origins of Blood Acetate in the Rat. Biochem. J. 166, 539–545. doi: 10.1042/bj1660539 597244 PMC1165038

[B15] Burger-van Paassen N. Vincent A. Puiman P. J. van der Sluis M. Bouma J. Boehm G.. (2009). The Regulation of Intestinal Mucin MUC2 Expression by Short-Chain Fatty Acids: Implications for Epithelial Protection. Biochem. J. 420, 211–219. doi: 10.1042/BJ20082222 19228118

[B16] Byrd A. L. Belkaid Y. Segre J. A. (2018). The Human Skin Microbiome. Nat. Rev. Microbiol. 16, 143–155. doi: 10.1038/nrmicro.2017.157 29332945

[B17] Carr Z. J. Van De Louw A. Fehr G. Li J. D. Kunselman A. Ruiz-Velasco V. (2018). Increased Whole Blood FFA2/GPR43 Receptor Expression Is Associated With Increased 30-Day Survival in Patients With Sepsis. BMC Res. Notes 11, 41. doi: 10.1186/s13104-018-3165-4 29338778 PMC5771199

[B18] Chambers E. S. Preston T. Frost G. Morrison D. J. (2018). Role of Gut Microbiota-Generated Short-Chain Fatty Acids in Metabolic and Cardiovascular Health. Curr. Nutr. Rep. 7, 198–206. doi: 10.1007/s13668-018-0248-8 30264354 PMC6244749

[B19] Chaudry A. N. Travers P. J. Yuenger J. Colletta L. Evans P. Zenilman J. M.. (2004). Analysis of Vaginal Acetic Acid in Patients Undergoing Treatment for Bacterial Vaginosis. J. Clin. Microbiol. 42, 5170–5175. doi: 10.1128/JCM.42.11.5170-5175.2004 15528711 PMC525245

[B20] Chemudupati M. Kenney A. D. Smith A. C. Fillinger R. J. Zhang L. Zani A.. (2020). Butyrate Reprograms Expression of Specific Interferon-Stimulated Genes. J. Virol. 94, 1–13. doi: 10.1128/JVI.00326-20 PMC7394905 32461320

[B21] Coll R. C. O'Neill L. A. (2010). New Insights Into the Regulation of Signalling by Toll-Like Receptors and Nod-Like Receptors. J. Innate Immun. 2, 406–421. doi: 10.1159/000315469 20505309

[B22] Corberand J. X. Laharrague P. F. Fillola G. (1989). Human Neutrophils Are Not Severely Injured in Conditions Mimicking Social Drinking. Alcohol Clin. Exp. Res. 13, 542–546. doi: 10.1111/j.1530-0277.1989.tb00374.x 2552861

[B23] Cummings J. H. Pomare E. W. Branch W. J. Naylor C. P. Macfarlane G. T. (1987). Short Chain Fatty Acids in Human Large Intestine, Portal, Hepatic and Venous Blood. Gut 28, 1221–1227. doi: 10.1136/gut.28.10.1221 3678950 PMC1433442

[B24] Dahlstrand Rudin A. Khamzeh A. Venkatakrishnan V. Basic A. Christenson K. Bylund J. (2021). Short Chain Fatty Acids Released by Fusobacterium Nucleatum Are Neutrophil Chemoattractants Acting *via* Free Fatty Acid Receptor 2 (FFAR2). Cell Microbiol. 23, e13348. doi: 10.1111/cmi.13348 33913592

[B25] Dalile B. Van Oudenhove L. Vervliet B. Verbeke K. (2019). The Role of Short-Chain Fatty Acids in Microbiota-Gut-Brain Communication. Nat. Rev. Gastroenterol. Hepatol. 16, 461–478. doi: 10.1038/s41575-019-0157-3 31123355

[B26] Eltzschig H. K. Carmeliet P. (2011). Hypoxia and Inflammation. N Engl. J. Med. 364, 656–665. doi: 10.1056/NEJMra0910283 21323543 PMC3930928

[B27] Fachi J. L. Secca C. Rodrigues P. B. Mato F. C. P. Di Luccia B. Felipe J. S.. (2020). Acetate Coordinates Neutrophil and ILC3 Responses Against C. Difficile Through FFAR2. J. Exp. Med. 217, 1–18. doi: 10.1084/jem.20190489 PMC7062529 31876919

[B28] Fanos V. Caboni P. Corsello G. Stronati M. Gazzolo D. Noto A.. (2014). Urinary (1)H-NMR and GC-MS Metabolomics Predicts Early and Late Onset Neonatal Sepsis. Early Hum. Dev. 90 Suppl 1, S78–S83. doi: 10.1016/S0378-3782(14)70024-6 24709468

[B29] Galvao I. Tavares L. P. Correa R. O. Fachi J. L. Rocha V. M. Rungue M.. (2018). The Metabolic Sensor GPR43 Receptor Plays a Role in the Control of Klebsiella Pneumoniae Infection in the Lung. Front. Immunol. 9, 142. doi: 10.3389/fimmu.2018.00142 29515566 PMC5826235

[B30] Godini R. Fallahi H. Ebrahimie E. (2018). Network Analysis of Inflammatory Responses to Sepsis by Neutrophils and Peripheral Blood Mononuclear Cells. PloS One 13, e0201674. doi: 10.1371/journal.pone.0201674 30086151 PMC6080784

[B31] Goncalves P. Araujo J. R. Di Santo J. P. (2018). A Cross-Talk Between Microbiota-Derived Short-Chain Fatty Acids and the Host Mucosal Immune System Regulates Intestinal Homeostasis and Inflammatory Bowel Disease. Inflammation Bowel Dis. 24, 558–572. doi: 10.1093/ibd/izx029 29462379

[B32] Gorbach S. L. Mayhew J. W. Bartlett J. G. Thadepalli H. Onderdonk A. B. (1976). Rapid Diagnosis of Anaerobic Infections by Direct Gas-Liquid Chromatography of Clinical Speciments. J. Clin. Invest. 57, 478–484. doi: 10.1172/JCI108300 1254729 PMC436673

[B33] Halestrap A. P. Meredith D. (2004). The SLC16 Gene Family-From Monocarboxylate Transporters (MCTs) to Aromatic Amino Acid Transporters and Beyond. Pflugers Arch. 447, 619–628. doi: 10.1007/s00424-003-1067-2 12739169

[B34] Hong Y. H. Nishimura Y. Hishikawa D. Tsuzuki H. Miyahara H. Gotoh C.. (2005). Acetate and Propionate Short Chain Fatty Acids Stimulate Adipogenesis *via* GPCR43. Endocrinology 146, 5092–5099. doi: 10.1210/en.2005-0545 16123168

[B35] Hoving L. R. Heijink M. van Harmelen V. van Dijk K. W. Giera M. (2018). GC-MS Analysis of Short-Chain Fatty Acids in Feces, Cecum Content, and Blood Samples. Methods Mol. Biol. 1730, 247–256. doi: 10.1007/978-1-4939-7592-1_17 29363078

[B36] Jiang L. Gulanski B. I. De Feyter H. M. Weinzimer S. A. Pittman B. Guidone E.. (2013). Increased Brain Uptake and Oxidation of Acetate in Heavy Drinkers. J. Clin. Invest. 123, 1605–1614. doi: 10.1172/JCI65153 23478412 PMC3613911

[B37] Jorth P. Turner K. H. Gumus P. Nizam N. Buduneli N. Whiteley M. (2014). Metatranscriptomics of the Human Oral Microbiome During Health and Disease. mBio 5, e01012–e01014. doi: 10.1128/mBio.01012-14 24692635 PMC3977359

[B38] Kamato D. Thach L. Bernard R. Chan V. Zheng W. Kaur H.. (2015). Structure, Function, Pharmacology, and Therapeutic Potential of the G Protein, Galpha/Q,11. Front. Cardiovasc. Med. 2, 14. doi: 10.3389/fcvm.2015.00014 26664886 PMC4671355

[B39] Karaki S. Mitsui R. Hayashi H. Kato I. Sugiya H. Iwanaga T.. (2006). Short-Chain Fatty Acid Receptor, GPR43, Is Expressed by Enteroendocrine Cells and Mucosal Mast Cells in Rat Intestine. Cell Tissue Res. 324, 353–360. doi: 10.1007/s00441-005-0140-x 16453106

[B40] Kedia-Mehta N. Finlay D. K. (2019). Competition for Nutrients and its Role in Controlling Immune Responses. Nat. Commun. 10, 2123. doi: 10.1038/s41467-019-10015-4 31073180 PMC6509329

[B41] Kendrick S. F. O'Boyle G. Mann J. Zeybel M. Palmer J. Jones D. E.. (2010). Acetate, the Key Modulator of Inflammatory Responses in Acute Alcoholic Hepatitis. Hepatology 51, 1988–1997. doi: 10.1002/hep.23572 20232292

[B42] Kim M. H. Kang S. G. Park J. H. Yanagisawa M. Kim C. H. (2013). Short-Chain Fatty Acids Activate GPR41 and GPR43 on Intestinal Epithelial Cells to Promote Inflammatory Responses in Mice. Gastroenterology 145, 396–406.e1-10. doi: 10.1053/j.gastro.2013.04.056 23665276

[B43] Knowles S. E. Jarrett I. G. Filsell O. H. Ballard F. J. (1974). Production and Utilization of Acetate in Mammals. Biochem. J. 142, 401–411. doi: 10.1042/bj1420401 4441381 PMC1168292

[B44] Krejner A. Bruhs A. Mrowietz U. Wehkamp U. Schwarz T. Schwarz A. (2018). Decreased Expression of G-Protein-Coupled Receptors GPR43 and GPR109a in Psoriatic Skin can be Restored by Topical Application of Sodium Butyrate. Arch. Dermatol. Res. 310, 751–758. doi: 10.1007/s00403-018-1865-1 30209581

[B45] Ladas S. Arapakis G. Malamou-Ladas H. Palikaris G. Arseni A. (1979). Rapid Diagnosis of Anaerobic Infections by Gas-Liquid Chromatography. J. Clin. Pathol. 32, 1163–1167. doi: 10.1136/jcp.32.11.1163 41850 PMC1145918

[B46] Laharrague P. Corberand J. Fillola G. Gleizes B. Gyrard E. Fontanilles A. M. (1985). [Effect of Ethanol on Human Polynuclear Neutrophils. *In Vitro* and *In Vivo* Study]. Ann. Med. Interne. (Paris) 136, 210–212. 4026107

[B47] Lam T. H. Verzotto D. Brahma P. Ng A. H. Q. Hu P. Schnell D.. (2018). Understanding the Microbial Basis of Body Odor in Pre-Pubescent Children and Teenagers. Microbiome 6, 213. doi: 10.1186/s40168-018-0588-z 30497517 PMC6267001

[B48] Le Poul E. Loison C. Struyf S. Springael J. Y. Lannoy V. Decobecq M. E.. (2003). Functional Characterization of Human Receptors for Short Chain Fatty Acids and Their Role in Polymorphonuclear Cell Activation. J. Biol. Chem. 278, 25481–25489. doi: 10.1074/jbc.M301403200 12711604

[B49] Louis P. Hold G. L. Flint H. J. (2014). The Gut Microbiota, Bacterial Metabolites and Colorectal Cancer. Nat. Rev. Microbiol. 12, 661–672. doi: 10.1038/nrmicro3344 25198138

[B50] Louis P. Young P. Holtrop G. Flint H. J. (2010). Diversity of Human Colonic Butyrate-Producing Bacteria Revealed by Analysis of the Butyryl-CoA:acetate CoA-Transferase Gene. Environ. Microbiol. 12, 304–314. doi: 10.1111/j.1462-2920.2009.02066.x 19807780

[B51] Lu R. Meng H. Gao X. Xu L. Feng X. (2014). Effect of non-Surgical Periodontal Treatment on Short Chain Fatty Acid Levels in Gingival Crevicular Fluid of Patients With Generalized Aggressive Periodontitis. J. Periodontal Res. 49, 574–583. doi: 10.1111/jre.12137 25340203

[B52] Lussu M. Camboni T. Piras C. Serra C. Del Carratore F. Griffin J.. (2017). (1)H NMR Spectroscopy-Based Metabolomics Analysis for the Diagnosis of Symptomatic E. Coli-Associated Urinary Tract Infection (UTI). BMC Microbiol. 17, 201. doi: 10.1186/s12866-017-1108-1 28934947 PMC5609053

[B53] Macfarlane S. Macfarlane G. T. (2003). Regulation of Short-Chain Fatty Acid Production. Proc. Nutr. Soc. 62, 67–72. doi: 10.1079/PNS2002207 12740060

[B54] Marshall D. D. Sadykov M. R. Thomas V. C. Bayles K. W. Powers R. (2016). Redox Imbalance Underlies the Fitness Defect Associated With Inactivation of the Pta-AckA Pathway in Staphylococcus Aureus. J. Proteome Res. 15, 1205–1212. doi: 10.1021/acs.jproteome.5b01089 26975873 PMC4875753

[B55] Maslowski K. M. Vieira A. T. Ng A. Kranich J. Sierro F. Yu D.. (2009). Regulation of Inflammatory Responses by Gut Microbiota and Chemoattractant Receptor GPR43. Nature 461, 1282–1286. doi: 10.1038/nature08530 19865172 PMC3256734

[B56] McNelis J. C. Lee Y. S. Mayoral R. van der Kant R. Johnson A. M. Wollam J.. (2015). GPR43 Potentiates Beta-Cell Function in Obesity. Diabetes 64, 3203–3217. doi: 10.2337/db14-1938 26023106 PMC4542437

[B57] Milligan G. Shimpukade B. Ulven T. Hudson B. D. (2017). Complex Pharmacology of Free Fatty Acid Receptors. Chem. Rev. 117, 67–110. doi: 10.1021/acs.chemrev.6b00056 27299848

[B58] Morrison D. J. Preston T. (2016). Formation of Short Chain Fatty Acids by the Gut Microbiota and Their Impact on Human Metabolism. Gut Microbes 7, 189–200. doi: 10.1080/19490976.2015.1134082 26963409 PMC4939913

[B59] Nadeem A. Ahmad S. F. Al-Harbi N. O. El-Sherbeeny A. M. Al-Harbi M. M. Almukhlafi T. S. (2017). GPR43 Activation Enhances Psoriasis-Like Inflammation Through Epidermal Upregulation of IL-6 and Dual Oxidase 2 Signaling in a Murine Model. Cell Signal 33, 59–68. doi: 10.1016/j.cellsig.2017.02.014 28212864

[B60] Nakajima A. Nakatani A. Hasegawa S. Irie J. Ozawa K. Tsujimoto G.. (2017). The Short Chain Fatty Acid Receptor GPR43 Regulates Inflammatory Signals in Adipose Tissue M2-Type Macrophages. PloS One 12, e0179696. doi: 10.1371/journal.pone.0179696 28692672 PMC5503175

[B61] Nohr M. K. Pedersen M. H. Gille A. Egerod K. L. Engelstoft M. S. Husted A. S.. (2013). GPR41/FFAR3 and GPR43/FFAR2 as Cosensors for Short-Chain Fatty Acids in Enteroendocrine Cells vs FFAR3 in Enteric Neurons and FFAR2 in Enteric Leukocytes. Endocrinology 154, 3552–3564. doi: 10.1210/en.2013-1142 23885020

[B62] Nuutinen H. Lindros K. Hekali P. Salaspuro M. (1985). Elevated Blood Acetate as Indicator of Fast Ethanol Elimination in Chronic Alcoholics. Alcohol 2, 623–626. doi: 10.1016/0741-8329(85)90090-4 4026986

[B63] Parada Venegas D. de la Fuente M. K. Landskron G. Gonzalez M. J. Quera R. Dijkstra G.. (2019). Corrigendum: Short Chain Fatty Acids (SCFAs)-Mediated Gut Epithelial and Immune Regulation and Its Relevance for Inflammatory Bowel Diseases. Front. Immunol. 10, 1486. doi: 10.3389/fimmu.2019.01486 31316522 PMC6611342

[B64] Paulus P. Jennewein C. Zacharowski K. (2011). Biomarkers of Endothelial Dysfunction: Can They Help Us Deciphering Systemic Inflammation and Sepsis? Biomarkers 16 Suppl 1, S11–S21. doi: 10.3109/1354750X.2011.587893 21707440

[B65] Peng G. S. Chen Y. C. Tsao T. P. Wang M. F. Yin S. J. (2007). Pharmacokinetic and Pharmacodynamic Basis for Partial Protection Against Alcoholism in Asians, Heterozygous for the Variant ALDH2*2 Gene Allele. Pharmacogenet. Genomics 17, 845–855. doi: 10.1097/FPC.0b013e3282609e67 17885622

[B66] Qiqiang L. Huanxin M. Xuejun G. (2012). Longitudinal Study of Volatile Fatty Acids in the Gingival Crevicular Fluid of Patients With Periodontitis Before and After Nonsurgical Therapy. J. Periodontal Res. 47, 740–749. doi: 10.1111/j.1600-0765.2012.01489.x 22594616

[B67] Ratajczak W. Ryl A. Mizerski A. Walczakiewicz K. Sipak O. Laszczynska M. (2019). Immunomodulatory Potential of Gut Microbiome-Derived Short-Chain Fatty Acids (SCFAs). Acta Biochim. Pol. 66, 1–12. doi: 10.18388/abp.2018_2648 30831575

[B68] Sadykov M. R. Thomas V. C. Marshall D. D. Wenstrom C. J. Moormeier D. E. Widhelm T. J.. (2013). Inactivation of the Pta-AckA Pathway Causes Cell Death in Staphylococcus Aureus. J. Bacteriol. 195, 3035–3044. doi: 10.1128/JB.00042-13 23625849 PMC3697545

[B69] Sanford J. A. O'Neill A. M. Zouboulis C. C. Gallo R. L. (2019). Short-Chain Fatty Acids From Cutibacterium Acnes Activate Both a Canonical and Epigenetic Inflammatory Response in Human Sebocytes. J. Immunol. 202, 1767–1776. doi: 10.4049/jimmunol.1800893 30737272 PMC7251550

[B70] Sanford J. A. Zhang L. J. Williams M. R. Gangoiti J. A. Huang C. M. Gallo R. L. (2016). Inhibition of HDAC8 and HDAC9 by Microbial Short-Chain Fatty Acids Breaks Immune Tolerance of the Epidermis to TLR Ligands. Sci. Immunol. 1. doi: 10.1126/sciimmunol.aah4609 28783689

[B71] Schlatterer K. Beck C. Schoppmeier U. Peschel A. Kretschmer D. (2021). Acetate Sensing by GPR43 Alarms Neutrophils and Protects From Severe Sepsis. Commun. Biol. 4, 928. doi: 10.1038/s42003-021-02427-0 34330996 PMC8324776

[B72] Sencio V. Barthelemy A. Tavares L. P. Machado M. G. Soulard D. Cuinat C.. (2020). Gut Dysbiosis During Influenza Contributes to Pulmonary Pneumococcal Superinfection Through Altered Short-Chain Fatty Acid Production. Cell Rep. 30, 2934–2947 e6. doi: 10.1016/j.celrep.2020.02.013 32130898

[B73] Shellito J. E. quan Zheng M. Ye P. Ruan S. Shean M. K. Kolls J. (2001). Effect of Alcohol Consumption on Host Release of Interleukin-17 During Pulmonary Infection With Klebsiella Pneumoniae. Alcohol Clin. Exp. Res. 25, 872–881. doi: 10.1111/j.1530-0277.2001.tb02293.x 11410724

[B74] Suckow A. T. Briscoe C. P. (2017). Key Questions for Translation of FFA Receptors: From Pharmacology to Medicines. Handb. Exp. Pharmacol. 236, 101–131. doi: 10.1007/164_2016_45 27873087

[B75] Tang C. Offermanns S. (2017). FFA2 and FFA3 in Metabolic Regulation. Handb. Exp. Pharmacol. 236, 205–220. doi: 10.1007/164_2016_50 27757760

[B76] Tan J. McKenzie C. Potamitis M. Thorburn A. N. Mackay C. R. Macia L. (2014). The Role of Short-Chain Fatty Acids in Health and Disease. Adv. Immunol. 121, 91–119. doi: 10.1016/B978-0-12-800100-4.00003-9 24388214

[B77] Todorovic V. Koko V. Lackovic V. Milin J. Varagic J. (1994). Effect of Chronic Alcohol Feeding on the Ultrastructure of Rat Peripheral Blood Neutrophils: A Morphometric Study. J. Stud. Alcohol 55, 239–248. doi: 10.15288/jsa.1994.55.239 8189745

[B78] Todorovic V. Koko V. Petakov M. Jovcic G. Stojanovic N. Bugarski D.. (1999). Cytochemical & Ultrastructural Alteration of Cytoplasmic Granules of Rat Peripheral Blood Neutrophils Induced by Chronic Alcoholism & Malnutrition. Indian J. Med. Res. 109, 105–114. 10489746

[B79] Tonetti M. Eftimiadi C. Damiani G. Buffa P. Buffa D. Botta G. A. (1987). Short Chain Fatty Acids Present in Periodontal Pockets may Play a Role in Human Periodontal Diseases. J. Periodontal Res. 22, 190–191. doi: 10.1111/j.1600-0765.1987.tb01565.x 2955096

[B80] Vinolo M. A. Ferguson G. J. Kulkarni S. Damoulakis G. Anderson K. Bohlooly Y. M.. (2011). SCFAs Induce Mouse Neutrophil Chemotaxis Through the GPR43 Receptor. PloS One 6, e21205. doi: 10.1371/journal.pone.0021205 21698257 PMC3115979

[B81] Waldecker M. Kautenburger T. Daumann H. Busch C. Schrenk D. (2008). Inhibition of Histone-Deacetylase Activity by Short-Chain Fatty Acids and Some Polyphenol Metabolites Formed in the Colon. J. Nutr. Biochem. 19, 587–593. doi: 10.1016/j.jnutbio.2007.08.002 18061431

[B82] Won H. I. Watson S. M. Ahn J. S. Endres J. L. Bayles K. W. Sadykov M. R. (2021). Inactivation of the Pta-AckA Pathway Impairs Fitness of Bacillus Anthracis During Overflow Metabolism. J. Bacteriol. 203 (9.e00660-20), 1–12. doi: 10.1128/JB.00660-20 PMC8092162 33593944

[B83] Yang W. Xiao Y. Huang X. Chen F. Sun M. Bilotta A. J.. (2019). Microbiota Metabolite Short-Chain Fatty Acids Facilitate Mucosal Adjuvant Activity of Cholera Toxin Through GPR43. J. Immunol. 203, 282–292. doi: 10.4049/jimmunol.1801068 31076530 PMC6581581

[B84] Yap Y. A. McLeod K. H. McKenzie C. I. Gavin P. G. Davalos-Salas M. Richards J. L.. (2021). An Acetate-Yielding Diet Imprints an Immune and Anti-Microbial Programme Against Enteric Infection. Clin. Transl. Immunol. 10, e1233. doi: 10.1002/cti2.1233 PMC7809703 33489123

